# Demand prediction of medical services in home and community-based services for older adults in China using machine learning

**DOI:** 10.3389/fpubh.2023.1142794

**Published:** 2023-03-16

**Authors:** Yucheng Huang, Tingke Xu, Qingren Yang, Chengxi Pan, Lu Zhan, Huajian Chen, Xiangyang Zhang, Chun Chen

**Affiliations:** ^1^School of Public Health and Management, Wenzhou Medical University, Wenzhou, Zhejiang, China; ^2^The State Key Laboratory of Cellular Stress Biology, Innovation Center for Cell Signaling Network, School of Life Sciences, Xiamen University, Xiamen, China; ^3^Center for Healthy China Research, Wenzhou Medical University, Wenzhou, Zhejiang, China

**Keywords:** home and community-based services, Andersen's behavioral model, Chinese Longitudinal Healthy Longevity Survey, demand prediction model, machine learning

## Abstract

**Background:**

Home and community-based services are considered an appropriate and crucial caring method for older adults in China. However, the research examining demand for medical services in HCBS through machine learning techniques and national representative data has not yet been carried out. This study aimed to address the absence of a complete and unified demand assessment system for home and community-based services.

**Methods:**

This was a cross-sectional study conducted on 15,312 older adults based on the Chinese Longitudinal Healthy Longevity Survey 2018. Models predicting demand were constructed using five machine-learning methods: Logistic regression, Logistic regression with LASSO regularization, Support Vector Machine, Random Forest, and Extreme Gradient Boosting (XGboost), and based on Andersen's behavioral model of health services use. Methods utilized 60% of older adults to develop the model, 20% of the samples to examine the performance of models, and the remaining 20% of cases to evaluate the robustness of the models. To investigate demand for medical services in HCBS, individual characteristics such as predisposing, enabling, need, and behavior factors constituted four combinations to determine the best model.

**Results:**

Random Forest and XGboost models produced the best results, in which both models were over 80% at specificity and produced robust results in the validation set. Andersen's behavioral model allowed for combining odds ratio and estimating the contribution of each variable of Random Forest and XGboost models. The three most critical features that affected older adults required medical services in HCBS were self-rated health, exercise, and education.

**Conclusion:**

Andersen's behavioral model combined with machine learning techniques successfully constructed a model with reasonable predictors to predict older adults who may have a higher demand for medical services in HCBS. Furthermore, the model captured their critical characteristics. This method predicting demands could be valuable for the community and managers in arranging limited primary medical resources to promote healthy aging.

## 1. Introduction

In recent decades, the aging population in China has emerged as a prominent social problem (1). According to the seventh population census, in 2020, 13.50% of the total population i.e., 190.64 million people living in China were 65 years or older (2). It is estimated that at this rate China will become a moderately aged society by 2030 (3) leading to considerable health problems, with 75.8% of the aging population suffering from at least one chronic disease (4). The World Health Organization (WHO) proposes healthy aging as a strategy to deal with aged societies (5); it thus advises providing older adults with integrated healthcare services. It emphasizes on the concept of bio-psycho-social health i.e., maintaining good physiological, psychological, and social health conditions in older adults (5).

Of the globally available aging care services (6–8), the three mainstream care services are family-based, home- and community-based, and elder care institutions. Due to differing national and cultural conditions, the advantages and limitations of the care services vary. Home and community-based services (HCBS) refer to individual-centered care provided by the community at home. HCBS not only retains the traditional form of caring but also reduces daily care and financial burdens for children (9), along with addressing the psychological (10, 11) and physical needs (11) of older adults.

HCBS evolved in Western countries in the 1980s and became widely popular in Europe (12), the USA (13), and Australia (14). HCBS takes care of people with different needs, such as patients with disability (15), depression (16) and dementia (17). In China, HCBS gained importance and support from the government in 2008 (18, 19). Moreover, supply intensity of HCBS among whole nation gradually increased from 2008 to 2018, which supply rates of all services doubled (20). Over time HCBS became the most appropriate care service for older adults in China (21). The 2018 Chinese Longitudinal Healthy Longevity Survey (CLHLS) classified services into the following four types, with each type having two sub-categories: (a). medical service including home visits and healthcare education, (b). daily life care service including personal care and daily shopping, (c). spiritual and cultural service including social and recreational activities and psychological consulting, and (d). mediation service including legal aid and neighborhood relations. Among all four services, medical services were in the highest demand from 2008 to 2018 (22, 23) and provisions of home visit and healthcare education were limited due to strained primary medical resources. Predicting demand for medical services could help managers in better management and targeted delivery of the service. Based on a 2014 national survey of older adults, using a logit model, a study explored the factors that influenced the demand for HCBS (24). Global research on unmet HCBS demand is scarce, and research predicting HCBS demand is lacking (25, 26). Former research has adopted classification trees to predict if older adults would use HCBS (27), even though there were deficiencies between demand and supply. Recently, HCBS was in high demand, but the lack of a complete and unified demand assessment system created an inability to convert potential into effective demand (28). Moreover, community managers lacked comprehensive and accurate supply planning, thus, contributing to a severe mismatch between demand and supply. Thus, suggesting the necessity of exploring methods to assess service demand and provide efficient and cost-effective HCBS (29). Predicting the demand for HCBS among older adults could help managers provide targeted services and formulate short- and long-term plans to address deficiencies. Traditional regression methods utilized in previous studies require independence of each variable and cannot resolve collinearity between the variables. Extant studies have concentrated on specific populations or certain factors, consequently failing to comprehensively grasp the demands of the whole population and critical characteristics. Machine learning can incorporate variables, produce accurate results with fewer constraints, and explore crucial characteristics. Thus, machine learning has been widely adopted to predict demands of healthcare services. For instance, Light Gradient Boosting Machine was conducted in ambulance demand prediction in Singapore; Long-Short Term Memory, a method based on Recurrent Neural Network, was utilized to predict home hospitalization demand of cancer palliative patients; and Extreme Gradient Boosting (XGboost) was applied in outpatient appointment demand prediction (30–32). During the Covid-19 pandemic, machine learning helped predict demands of ICU, ventilator, and length of hospital stays (33).

Hence, to understand the demand for medical services in HCBS more comprehensively, Andersen's behavioral model of health service could be employed to bridge feature selection and initial feature selection as well as machine learning model fitting. Andersen's behavioral model of health service use was proposed in 1968 and subsequently modified several times. It is widely acknowledged and applied in health-related services, such as medical costs, healthcare utilization, and drug use. It is used to determine the factors that influence health service use at different levels, as well as the variables that could be more logical, diverse, and specific (26, 34–38). Andersen's behavioral model contains multiple domains of an individual: predisposing, enabling, need, and behavior. Each domain is associated with the outcome of demand for healthcare. Predisposing factors generally describe socio-demographic characteristics; enabling factors represent personal healthcare acquirement; need factors manifest self-cognition of a health condition; and behavior factors reflect lifestyle related to their physical, mental, and social health (39).

As medical services in HCBS had the highest demand (21) and a significant positive influence on health and chronic diseases (40, 41), this study aimed to identify the best model to predict demand for medical services in HCBS among older adults in China in 2018 and explore the most critical characteristics of older adults requiring the services. We hope that the findings of this study would help in increasing efficiency in matching the demand and supply of medical services in HCBS, considering the characteristics of older adults, and, thus, contribute to healthy aging.

## 2. Methods

### 2.1. Data sources and sample

This study used the 2018 CLHLS (*n* = 15,874) conducted by the Peking University Center for Healthy Aging and Family Studies and the China Mainland Information Group, every 3 years since 1998 (22).

Respondents in CLHLS were sampled randomly from households in half of the counties and cities across 23 provinces in mainland China. Instruments used for data collection were international questionnaires, interviews, basic physical capacity tests, and physical examinations. Former researchers demonstrated that the details of sample design and data quality were excellent (42). After excluding 3,933, participants younger than 65 years and/or those lacking information about the home and community-based medical services, 15,312 participants were included in the final data analysis.

### 2.2. Outcome variable: Demand for medical services in HCBS

Demand for medical services of HCBS was evaluated using two questions: “Do you expect your community to provide home visit services?” and “Do you expect your community to provide healthcare education services?” The expectation of one or more medical services was considered as a demand for HCBS. In case of no services expected, it was considered as no demand for medical services in HCBS.

### 2.3. Predictors and feature selection

We included a broad range of candidate predictors. Based on Andersen's behavioral model, the predictors were divided into predisposing, enabling, need, and behavior factors (34, 35). This model was proposed in 1968 and subsequently modified several times. The model is widely acknowledged and applied in the field of health-related services, such as medical costs, self-medication, and drug use, to determine influencing factors of health service use (36, 43).

Predisposing factors included demographic characteristics that may affect requirements for medical services. Factors included gender (male or female), age (65–79 years or ≥80 years), an education level (literate or illiterate), marital status (married or unmarried), and residence (rural, town, or urban). Enabling factors included individual characteristics that may affect requirements for medical services in HCBS, such as self-rated income level (low or high), pension (yes or no), social insurance (yes or no), living conditions (live with families, live alone, or live in care institution). Need factors included individual health status, such as chronic diseases (yes or no), activities of daily living (ADL) (good or bad), cognitive function (good or bad), and self-rated health (SRH) (good, fair, or poor). Behavioral factors included daily actions and habits that could affect an individual's physiological, mental, and social health, such as smoking (yes or no), alcohol consumption (yes or no), exercising (yes or no), and socializing (yes or no).

### 2.4. Statistical analysis

Statistical analyses were performed using the Scikit-Learn package (version 1.1.2) in Python (version 3.9) (44). Scikit-Learn is a wrapper technique; it was used to apply models to the data, which were randomly split into independent training, testing sets, and validation sets at a ratio of 6:2:2.

#### 2.4.1. Processing of missing values

To minimize the chance of bias owing to imputation, variables with more than 20% of information were abandoned to acquire reasonable performances. The ultimate variables included were imputed by the “MICE” package in R studio 4.1.2, applying “missForest” multivariate iterative random forest (“RF” method) imputation algorithm with five iterations and 100 estimators to obtain the least variant datasets compared to the original one.

#### 2.4.2. Synthetic minority oversampling technique

Lack of demand for HCBS medical services was low probability attitude resulting in an imbalanced dataset i.e., adults not requiring medical services while using HCBS were less prevalent than the others. The imbalanced data was a challenge for machine learning, as the sample size of older adults without demand was small. Furthermore, a strong bias toward the majority class is evident while evaluating the classification model, leading to sub-optimal performances. To resolve the issue, we applied Synthetic Minority Oversampling Technique (SMOTE), a statistical technique proposed by Chawla et al. (45). SMOTE generates virtual replicates from the existing minority class, thus expanding the number of minority samples in the datasets (45). SMOTE algorithm has been widely applied to process imbalance data in medical research and generally performs reasonable results with machine learning (46–48).

#### 2.4.3. Machine learning methods

We applied five machine learning methods, including single models and ensemble models. These were: logistic regression (LR), LR with lasso regularization, support vector machine (SVM), random forest (RF), and extreme gradient boosting (XGboost). The outcome variable in this study was binary, that is, irrespective of whether older adults in China needed medical services in HCBS, all selected five models were widely applied in binary outcome prediction with great performances (46, 49, 50). We compared their ability to predict demand for medical services in HCBS.

##### 2.4.3.1. Logistic regression

Logistic regression (LR) is a kind of general linear model. The model has a potential assumption that the outputs or the results conform to the Bernoulli distribution with parameter p. Parameter p is the probability of a positive result (in our case, the probability of demand for medical services in HCBS among older Chinese adults). Moreover, Logistic regression does demands rigorously for number of features and samples, and it could be applied in different populations (51). Parameters for Logistic regression used in this study are default in the Scikit-Learn package.

##### 2.4.3.2. LR with LASSO regularization

LASSO regression is a member of the general linear model family. It is an approach to conduct variable selection and regularization while fitting the regression model. By setting parameter α to penalize the original linear model, LASSO regularization deals with the highly correlated variables to minimize the possibilities of over-fit; this automatically drops unnecessary covariates and preserves the most critical variables. Several studies have demonstrated that lasso regression has many ideal properties that can be used to enhance LR model's performance while including more covariates and the ability to predict outcomes in other populations. In this research, we selected the parameter (α = 0.01) to penalize large coefficients that resulted in a maximum correct classification rate and the best model performance (52, 53).

##### 2.4.3.3. Support vector machine

Support Vector Machine (SVM) is a manually controlled classification algorithm, by the statistical theory. The working principle of SVM is to create a decision boundary, based on the definition of the hyperplane, that could separate the two categories from each other in an accurate split method. There are four widely adopted kernel functions in SVM: linear, sigmoid, radial basis (RBF), and polynomial. RBF kernel was applied in this study to construct the hyperplane due to the number of features and total samples (54–56).

##### 2.4.3.4. Random forest

Random Forest (RF) is a typical ensemble algorithm consisting of a series of decision trees as its basic unit using the Bagging method. Each tree randomly selects training samples and sample characteristics from the group and returns them to the original datasets to ensure that the amount of training samples is the same in each model. Due to these two features, the set of constructed decision trees contains abundant information for classification. To analysis the ultimate result, each decision tree is accessed to the final decision for a reliable result. Based on the majority voting on all decision trees, each sample is classified into two classes. We adopted 1,000 estimators with defaults for other parameters to assess the model and explore the features of older adults with/without demand toward medical services in HCBS (57, 58).

##### 2.4.3.5. Extreme gradient boosting (XGboost)

XGboost classification algorithm is an ensemble algorithm of a decision tree, adopting boosting sampling method. It is an enhanced Gradient Boosting algorithm that reduces the probability of over-fit by regularizing the loss function and improves algorithm accuracy by approaching the real loss during each gradient process. In addition, XGboost possesses the ability to directly handle the encoded categorical variables. Therefore, we set 1,000 decision trees, with other parameters as defaults, to predict outcomes of demand for HBCS medical services and explore the importance of individual features (59, 60).

### 2.5. Model assessment

To assess the outcomes of each machine learning model, we observed the value of area under the receiver operating curve (ROC; AUC), sensitivity [Eq. (1)], specificity [Eq. (2)], accuracy [Eq. (3)], and balanced accuracy [Eq. (4)]. Moreover, to obtain a further understanding of the contribution of each predictor to the machine learning model and to explore the effect of individual characteristics on the demand of HCBS medical services, we calculated the importance of variables in the RF and XGboost models for each result.


(1) $$\begin{array}{lll} Sensitivity & = & \frac{\text{TP}}{\text{TP}\;+\;\text{FN}} \end{array}$$

(2) $$\begin{array}{lll} Specificity & = & \frac{\text{TN}}{\text{TN}\;+\;\text{FP}} \end{array}$$

(3) $$\begin{array}{lll} Accuracy & = & \frac{\text{TN}\;+\;\text{TP}}{\text{TN}\;+\;\text{TP}\;+\;\text{FN}\;+\;\text{FP}} \end{array}$$

(4) $$\begin{array}{l} Balanceaccuracy=\frac{2\;*\;\text{Sensitivity }*\text{ Specificity}}{\text{Sensitivity }+\;\text{Specificity}} \end{array}$$

True positives (TP) and True negatives (TN) indicated older adults who were identified as with and without the demand for HCBS healthcare, respectively; False positives (FP) and false negatives (FN) indicated older adults who were inaccurately identified as with and without the demand for healthcare HCBS, respectively.

## 3. Results

As shown in Table 1, 15,312 participants were included in this study, but only 13,244 older adults demanded medical services in HCBSs, thus, the demand rate was 86.48%. We also analyzed crude and adjusted odds ratio for older adults who demanded medical services in HCBS using single and multiple variable binary logistic regression. The analysis demonstrates that illiterate older adults had higher possibilities (adjusted OR = 1.21; 95% CI: 1.07–1.36) of requiring medical services in HCBS. Compared to the urban older adults, older adults living in town (adjusted OR = 1.95; 95% CI: 1.70–2.20) and rural (adjusted OR = 1.92; 95% CI: 1.68–2.16) areas had higher demand for the service. Among enabling factors, the older adults not having social insurance (adjusted OR = 1.20; 95% CI: 1.09–1.32), needed more medical services provided by HCBS. Moreover, fair self-rated health status (adjusted OR = 1.18; 95% CI: 1.06–1.31) increased the possibility of demand for services among older adults. The results also indicate that the regular exercising group (adjusted OR = 1.26; 95% CI: 1.13–1.40) and older adults dislike socializing (adjusted OR = 0.85; 95% CI: 0.73–0.99) and had lower demand for medical services in HCBS.

**Table 1:** Characteristics and odds ratio of older adults with demands of medical services provided by HCBS in CLHLS 2018.

**Predictors**		**All *N* (%)**	**With demand *N* (%)**	**Without demand *N* (%)**	**Crude OR (95% CI)**	**Adjusted OR (95% CI)**
Overall	15,312	13,242 (86.48%)	2,070 (13.53%)			
**Predisposing factors**						
Gender	Male	6,687 (43.67%)	5,788 (43.71%)	899 (13.44%)	Ref	Ref
	Female	8,625 (56.33%)	7,454 (56.29%)	1,171 (13.57%)	0.99 (0.90, 1.09)	0.93 (0.83, 1.05)
Age	65–79	5,213 (34.05%)	4,549 (34.35%)	664 (32.08%)	Ref	Ref
	≥80	10,099 (65.95%)	8,693 (65.65%)	1,406 (67.92%)	**0.90 (0.82, 0.99)** ^***^	0.91 (0.80, 1.03)
Education	Non-illiterate	7,707 (50.33%)	6,759 (51.04%)	948 (45.80%)	Ref	Ref
	Illiterate	7,605 (49.67%)	6,483 (48.96%)	1,122 (54.20%)	**1.23 (1.12, 1.35)** ^***^	**1.21 (1.07, 1.36)** ^***^
Marital status	Single	9,283 (60.63%)	7,984 (60.29%)	1,299 (62.75%)	Ref	Ref
	Married	6,029 (39.37%)	5,258 (39.71%)	771 (37.25%)	**0.90 (0.82, 0.99)** ^*^	0.91 (0.80, 1.03)
Residence	Urban	3,454 (22.56%)	2,762 (20.86%)	692 (33.43%)	Ref	Ref
	Town	5,073 (33.13%)	4,488 (33.89%)	585 (28.26%)	**1.92 (1.71, 2.17)** ^***^	**1.95 (1.70, 2.20)** ^***^
	Rural	6,785 (44.31%)	5,992 (45.25%)	793 (38.31%)	**1.89 (1.69, 2.12)** ^***^	**1.92 (1.68, 2.16)** ^***^
**Enabling factors**						
Income level	Low	1,671 (10.91%)	1,467 (11.08%)	204 (9.86%)	Ref	Ref
	High	13,641 (89.09%)	11,775 (88.92%)	1,866 (90.14%)	1.14 (0.98, 1.33)	1.01 (0.86, 1.18)
Pension	Yes	9,911 (64.73%)	8,568 (64.70%)	1,343 (64.88%)	Ref	Ref
	No	5,401 (35.27%)	4,674 (35.30%)	727 (35.12%)	0.99 (0.90, 1.09)	0.96 (0.87, 1.06)
Social insurance	Yes	8,614 (56.26%)	7,481 (56.49%)	1,133 (54.73%)	Ref	Ref
	No	6,698 (43.74%)	5,761 (43.51%)	937 (45.27%)	1.08 (0.98, 1.18)	**1.20 (1.09, 1.32)** ^***^
Living status	Family	12,315 (80.43%)	10,658 (80.49%)	1,657 (80.05%)	Ref	Ref
	Alone	2,433 (15.895)	2,109 (15.93%)	324 (15.65%)	1.01 (0.89, 1.15)	1.02 (0.88, 1.17)
	Institution	564 (3.68%)	475 (3.59%)	89 (4.30%)	0.83 (0.66, 1.05)	1.09 (0.86, 1.39)
**Need factors**						
Chronic disease	Yes	2,635 (17.21%)	2,271 (17.15%)	364 (17.58%)	Ref	Ref
	No	12,677 (82.79%)	10,971 (82.85%)	1,706 (82.42%)	0.97 (0.86, 1.10)	0.91 (0.81, 1.04)
ADL	Yes	4,305 (28.12%)	3,695 (27.90%)	610 (29.47%)	Ref	Ref
	No	11,007 (71.88%)	9,547 (72.10%)	1,460 (70.53%)	0.93 (0.84, 1.03)	1.03 (0.91, 1.17)
Cognitive loss	Yes	5,860 (38.27%)	5,047 (38.11%)	813 (39.28%)	Ref	Ref
	No	9,452 (61.73%)	8,195 (61.89%)	1,257 (60.72%)	0.95 (0.87, 1.05)	0.92 (0.81, 1.04)
SRH	Good	7,106 (46.41%)	6,067 (45.82%)	1,039 (50.19%)	Ref	Ref
	Fair	5,987 (39.10%)	5,254 (39.68%)	733 (35.41%)	**1.23 (1.11, 1.36)** ^***^	**1.18 (1.06, 1.31)** ^***^
	Bad	2,219 (14.49%)	1,921 (14.51%)	298 (14.40%)	1.10 (0.96, 1.27)	1.06 (0.92, 1.23)
**Behavior factors**						
Smoking	Yes	2,228 (14.55%)	1,933 (14.60%)	295 (14.25%)	Ref	Ref
	No	13,084 (85.45%)	11,309 (85.40%)	1,775 (85.75%)	0.97 (0.85, 1.11)	1.05 (0.91, 1.21)
Alcohol drinking	Yes	2,138 (13.96%)	1,848 (13.96%)	290 (14.01%)	Ref	Ref
	No	13,174 (86.04%)	11,394 (86.04%)	1,780 (85.99%)	1.01 (0.88, 1.15)	1.04 (0.90, 1.21)
Exercising	Yes	4,569 (29.84%)	3,839 (28.99%)	730 (35.27%)	Ref	Ref
	No	10,743 (70.16%)	9,403 (71.01%)	1,340 (64.73%)	**1.33 (1.21, 1.47)** ^***^	**1.26 (1.13, 1.40)** ^***^
Socializing	Yes	1,987 (12.98%)	1,708 (12.90%)	279 (13.48%)	Ref	Ref
	No	13,325 (87.02%)	11,534 (87.10%)	1,791 (86.52%)	1.05 (0.92, 1.21)	**0.85 (0.73, 0.99)** ^*^

Boldface indicated statistical significance (^*^p < 0.05; ^**^p < 0.01; ^***^p < 0.001).

The confusion metrics and the performance metrics shown in Table 2 illustrate the five machine learning methods in Models I-IV. LR served as the benchmark baseline with the AUC of 0.57, 0.59, 0.63, and 0.66 in Models I–IV, respectively. Lasso had a similar AUC as LR in Models I and IV. SVM had slightly higher AUC of 0.57, 0.60, 0.63, and 0.66, respectively. The AUC of RF (0.57, 0.61, 0.71, and 0.77) and XGboost (0.57, 0.61, 0.70, and 0.76) were higher than the AUC of the other machine learning methods in Models I-IV. Furthermore, RF and XGboost performed best in terms of sensitivity, specificity, accuracy, and balance in Model IV. The addition of need factors to Model II changed it to Model III, and it resulted in a greater change in AUC. This change could predict that need factors may have the greatest impact on the demand for medical services in HCBSs.

**Table 2:** Performance of machine learning models in prediction of the older adults' demand for medical services provided by HCBS in CLHLS 2018.

	**Classifier**	**AUC**	**TP/TN/FP/FN**	**Sensitivity (%)**	**Specificity (%)**	**Accuracy (%)**	**Balanced accuracy (%)**
Model I	LR	0.571 (0.555,0.586)	2076/891/1768/563	78.67 (77.10,80.23)	33.51 (31.71,35.30)	56.00 (54.68,57.35)	64.04 (62.88,65.21)
	LASSO	0.567 (0.552,0.583)	2076/891/1768/563	78.67 (77.10,80.23)	33.51 (31.71,35.30)	56.00 (54.68,57.35)	64.04 (62.88,65.21)
	SVM	0.570 (0.555,0.586)	2076/891/1768/563	78.67 (77.10,80.23)	33.51 (31.71,35.30)	56.00 (54.68,57.35)	64.04 (62.88,65.21)
	RF	0.568 (0.553,0.583)	1875/1100/1559/764	71.05 (69.32,72.78)	41.37 (39.50,43.24)	56.15 (54.83,57.50)	61.75 (60.53,62.97)
	XGboost	0.568 (0.546,0.577)	1891/1082/1577/748	71.66 (69.94,73.38)	40.69 (38.82,42.56)	56.12 (54.79,57.46)	61.93 (60.71,63.15)
Model II	LR	0.594 (0.579,0.609)	1679/1361/1298/960	63.62 (61.79,65.46)	51.18 (49.29,53.09)	57.38 (56.05,58.71)	59.79 (58.51,61.08)
	LASSO	0.594 (0.577,0.607)	1763/1252/1407/876	66.81 (65.01,68.60)	47.09 (45.19,48.98)	56.91 (55.58,58.24)	60.70 (59.44,61.96)
	SVM	0.602 (0.587,0.618)	2076/891/1768/563	78.67 (77.10,80.23)	33.51 (31.72,35.30)	56.00 (54.68,57.35)	64.04 (62.88,65.21)
	RF	0.615 (0.600,0.630)	1568/1689/970/1071	59.42 (57.54,61.29)	63.52 (61.69,65.35)	61.48 (60.18,62.80)	60.58 (59.24,61.91)
	XGboost	0.613 (0.597,0.627)	1587/1658/1001/1052	60.14 (58.27,62.00)	62.35 (60.51,64.20)	61.25 (59.95,62.57)	60.72 (59.40,62.05)
Model III	LR	0.630 (0.616,0.645)	1664/1481/1178/975	63.05 (61.21,64.90)	55.70 (53.81,57.59)	59.36 (58.04,60.69)	60.72 (59.42,62.01)
	LASSO	0.626 (0.611,0.641)	1658/1489/1170/981	62.83 (60.98,64.67)	56.00 (54.11,57.88)	59.40 (58.08,60.72)	60.65 (59.36,61.95)
	SVM	0.629 (0.614,0.644)	1741/1412/1247/898	65.97 (64.16,67.78)	53.10 (51.20,55.00)	59.51 (58.20,60.85)	61.88 (60.61,63.15)
	RF	0.712 (0.698,0.726)	1707/2050/609/932	64.68 (62.86,66.51)	77.10 (75.50,78.69)	70.91 (69.70,72.15)	68.90 (67.61,70.19)
	XGboost	0.697 (0.682.0.710)	1748/1940/719/891	66.24 (64.43,68.04)	72.96 (71.27,74.65)	69.61 (68.39,70.86)	68.47 (67.19,69.74)
Model IV	LR	0.656 (0.641,0.671)	1681/1540/1119/958	63.70 (61.86,65.53)	57.92 (56.04,59.79)	60.80 (59.48,62.11)	61.81 (60.52,63.10)
	LASSO	0.652 (0.637,0.667)	1659/1557/1102/980	62.86 (61.02,64.71)	58.56 (56.68,60.43)	60.70 (59.39,62.02)	61.44 (60.15,62.74)
	SVM	0.659 (0.645,0.674)	1737/1502/1157/902	65.82 (64.01,67.63)	56.49 (54.60,58.37)	61.14 (59.84,62.46)	62.79 (61.51,64.06)
	RF	0.773 (0.761,0.786)	1881/2191/444/781	70.66 (68.93,72.39)	83.15 (81.72,84.58)	76.87 (75.74,78.01)	75.44 (74.24,76.63)
	XGboost	0.758 (0.745,0.771)	1801/2182/452/862	67.63 (65.85,69.41)	82.84 (81.40,84.28)	74.16 (72.98,75.33)	72.09 (70.83,73.34)

Model I included predisposing factors; Model II included predisposing factors and enabling factors; Model III included predisposing factors, enabling factors, and need factors; Model IV included predisposing factors, enabling factors, need factors, and behavior factors.

**Figure 1:**
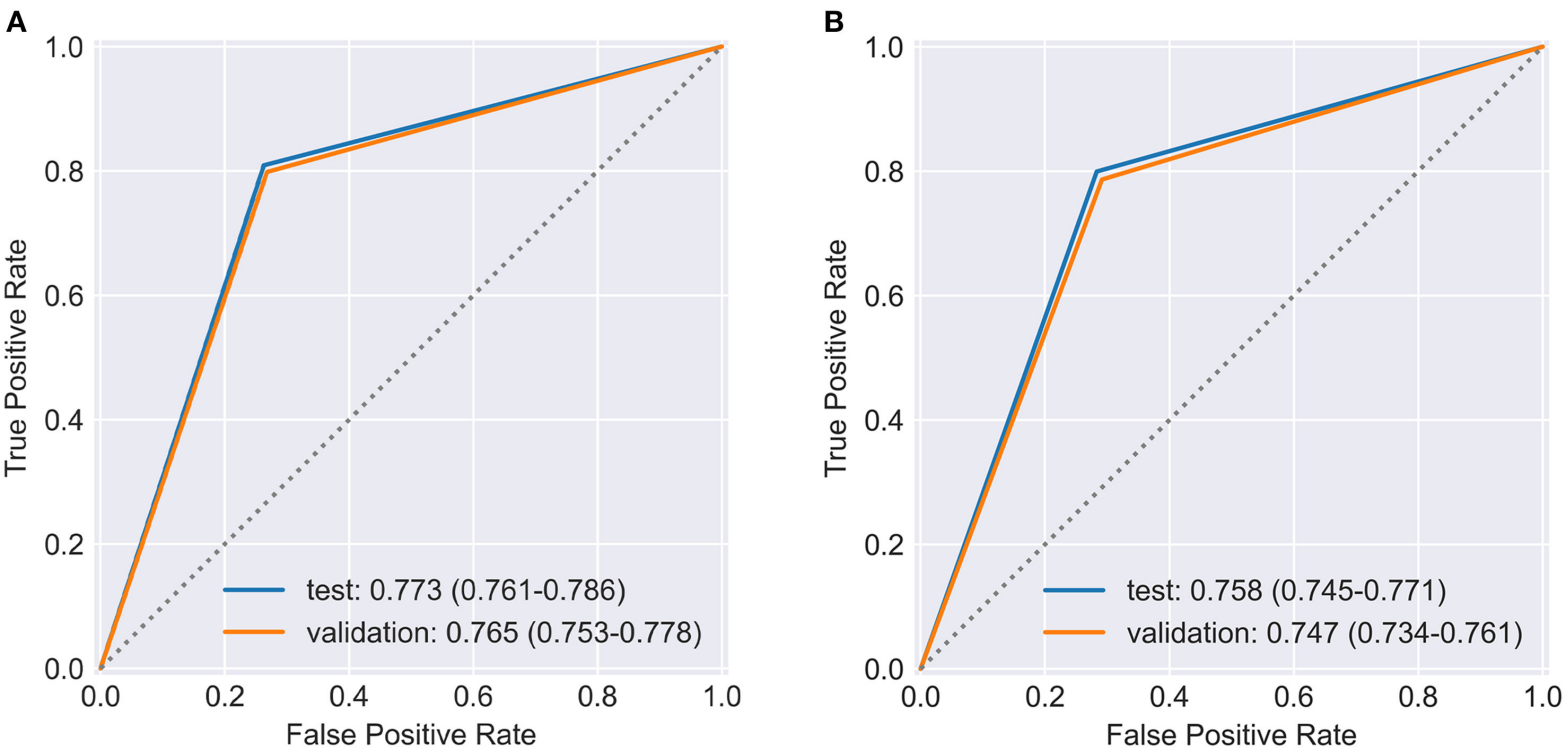
ROC and AUC performed by **(A)** RF and **(B)** XGboost in Model IV for both testing set, and validation set.

To evaluate the stability of Model IV, 20% of the total samples were separated, as the validation set, to examine if the models were over-fitted in the RF and XGboost. Figure 1A displays ROCs of Model IV fitted by RF, whose AUC did not show a significant difference between the test set and the validation set. In Figure 1B ROCs were produced by XGboost, which produced robust results in the validation set. Both models fitted by all four factors of Andersen's behavioral model as presented in Table 3 performed steady results to predict the demand for medical services in HCBS compared to the test set of Model IV in Table 2.

**Table 3:** Validation of RF and XGboost in Model IV.

**Model IV validation set**	**Classifier**	**AUC**	**TP/TN/FP/FN**	**Sensitivity (%)**	**Specificity (%)**	**Accuracy (%)**	**Balanced accuracy (%)**
	RF	0.765 (0.753,0.778)	1896/2139/479/783	70.77 (69.05,72.50)	81.70 (80.22,83.18)	76.18 (75.02,77.32)	75.03 (73.84,76.22)
	XGboost	0.747 (0.734,0.761)	1768/2160/480/889	66.54 (64.75,68.33)	81.82 (80.35,83.29)	74.16 (72.98,75.33)	72.09 (70.83,73.36)

**Figure 2:**
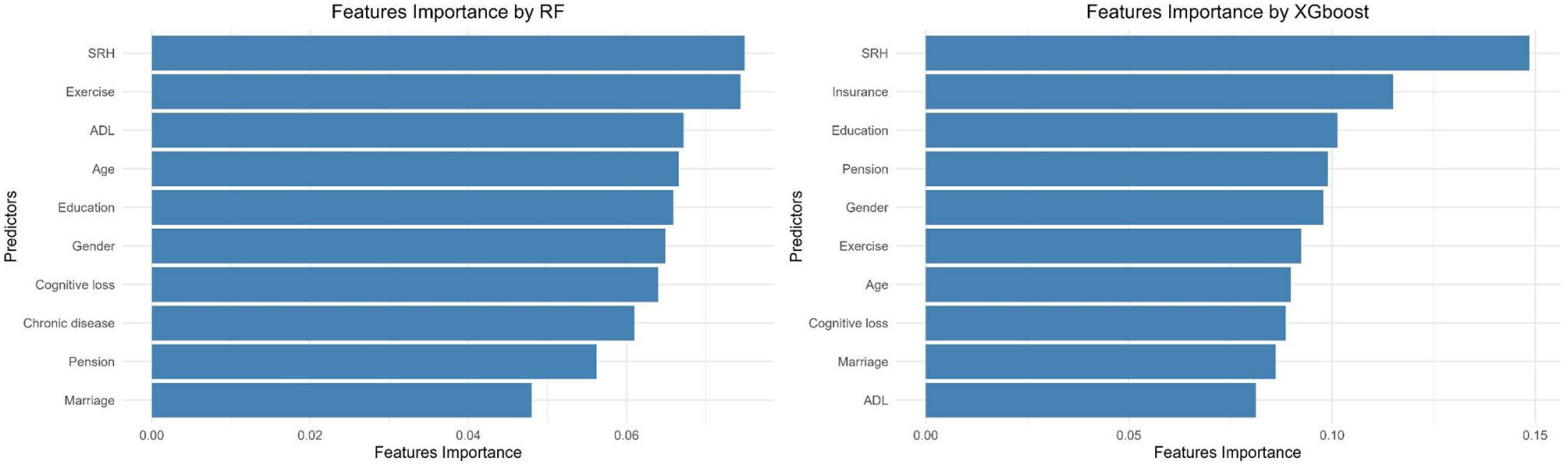
The most important features of the older adults, who demanded for medical services provided by HCBS in CLHLS 2018.

Figure 2 shows the importance of the predictors in the RF and XGboost. In the RF method SRH, exercise, ADL, age, education, and gender were the most important predictors of the demand for medical services in HCBS. Variable importance produced by XGboost demonstrated that SRH, social insurance, education, pension, gender, and exercise were the most critical features.

## 4. Discussion

To the best of our knowledge, this is the first research to predict the demand for medical services in HCBS among older adults in China using national representative data, CLHLS 2018, and including demographic, social, economic, health, and other parameters.

Although the demand proportion for healthcare services was relatively high worldwide (61, 62), our study revealed that it was higher in China. Along with the growing life expectancy, the average age continues to increase in China (18). As people age, their need for medical services increases (24, 63). Consequently, the demand for medical services provided by HCBS was high from 2008 to 2018, above 80%, with an upward trend. Moreover, with the change in the current family structure and fast-paced social life, the traditional family-based care modes have lost significance in promoting life satisfaction among older adults (64, 65). Hence, more empty-nest older adults who lived alone failed to get timely treatment (64). Additionally, a large number of older adults suffered from chronic diseases, such as hypertension, diabetes, and respiratory diseases that required daily medical monitoring to ensure older adults remain in normal living conditions (66).

Some studies successfully adopted traditional regression methods (24); however, deficiencies in traditional methods, which requires absolute independence among the variables, could lead to information loss during variable selection. Moreover, demand for medical services provided by HCBS had large imbalances, resulting in higher sensitivity and accuracy but lower specificity. Therefore, it was impractical to use, as only ~15% of the older adults did not need medical services in HCBS. As higher specificity was necessary to predict the group without need, utilizing SMOTE solved this issue; the AUC was higher for specificity (83.15% in RF and 82.84% in XGboost among Model IV). The performance of SMOTE resulted in better-fit results and produced robust data without missing samples, thus, creating a more practical model to predict older adults with and without need.

Machine learning models could include variables with fewer constraints, enabling the models to confront the presence of high dimensions and correlated predictors. Thus, they are a widely acknowledged and adapted method in exploring influencing factors of health-related services. HCBS is an integrated care service, covering the multilevel and diversified demands of older adults; therefore, by using the four factors in Andersen's behavioral model it was possible to explore the critical features above reasonable theoretical basis. The AUC and accuracy of RF and XGboost were increased sharply after including need factors. While all four factors were included in the machine learning models, the AUC of the five models was above 0.60, and RF and XGboost showed good model fit. The AUC of RF was beyond 0.75, demonstrating the feasibility of predicting the demands of older adults for medical services in HCBS, based on Andersen's behavioral model and machine learning methods. With high specificity, the model could filter the people who were more likely to have no demand for medical services in HCBS temporally. This would help decision-makers to provide older adults in urgent demand with targeted care in situations with limited resources. To examine robustness, the performance of the validation set proved the performances of these two models were not over-fitted.

Using Andersen's behavioral model, combined with Logistic regression and estimating the contribution of each variable in machine learning models, we further confirmed that self-rated health was the most significant feature to predict if older adults needed medical services in HCBS. The present research illustrated that health conditions had a direct influence on medical services in HCBS, which confirmed the results that SRH had the highest importance in predicting if older adults had demand (24). Moreover, the aged population with good health had a stronger demand for medical services provided by HCBS (67, 68). Previous research demonstrated that older adults in bad health went to the hospital and looked for more exhaustive medical services (69) whereas older adults with good health might not have urgent demand. Furthermore, there was strong evidence that confirmed chronic disease was a significant risk factor for poor SRH rate. These results could enable the community to provide medical services preferentially (70, 71).

Furthermore, exercise and education played important roles in demand. Illiterate people aged >65 years had lower health literacy levels (72, 73). Therefore, they may require healthcare education services more urgently (74). Participants who rarely exercised were more likely to gain weight and have worse health status. Appropriate exercise could meet the requirement of the bio-psycho-social medical model, by facilitating metabolism in older adults, obtaining a sense of happiness, and getting the chance to meet friends who share the same hobby (75, 76). Therefore, older adults who do not exercise may need medical services in HCBS more than those who exercise regularly (77).

These findings indicate that the characteristics of older adults should be considered to narrow the gap between supply and demand. Communities could (a) make efforts to focus on older adults with good health, (b) provide health education on conditions like hypertension, diabetes, and stroke, to promote health literacy in the neighborhood, and (c) propose targeted measures to encourage older adults to exercise, based on their abilities, and offer periodical home medical visits to monitor their health condition.

Andersen's behavioral model and machine learning could help managers and governments construct a complete and unified demand assessment system, which could also be extrapolated to other types of demands. This would enable HCBS to narrow the supply-demand gap and improve management efficiency and cost-effectiveness. Ultimately, this would promote healthy aging by providing more effective services.

## 5. Limitation

This study has some limitations. Firstly, we only adopted data from the 2018 CLHLS to predict demand for medical services provided by HCBS, thus, this cross-sectional data could not explore causality between demand and predictors. Second, the CLHLS provided national representative data. Previous research indicated that the supply situation and intensity of HCBS in China vary significantly temporally and spatially. This regional variance may increase the supply and demand mismatch and affect the information for the use of HCBS among older adults. Simultaneously, including all predictors as factor variables could lead to information loss in estimating the contribution of individual variables. Furthermore, this study included home medical visits and healthcare education as medical services. As interactions between these two services are possible, only extensive characteristic ranges could be determined to identify demand. As, HCBS included four types of services only, hence, to construct an assessment system, further research on demands predictions for other services is required.

## 6. Conclusion

This study adapted machine learning to predict the demand for medical services in HCBS using the 2018 CLHLS data based on Andersen's behavioral model. Andersen's behavioral model combined with machine learning successfully constructed a model with reasonable predictors and captured critical characteristics in older adults, who may have higher demand. This method predicting demands could be valuable for the community and decision-makers in arranging limited primary medical resources to promote healthy aging. Future empirical research should examine the models and conduct a longitudinal study to explore the causation between demand and individual characteristics.

## Data availability statement

Publicly available datasets were analyzed in this study. This data can be found here: https://opendata.pku.edu.cn/dataset.xhtml?persistentId=doi:10.18170/DVN/WBO7LK.

## Ethics statement

The studies involving human participants were reviewed and approved by Research Ethics Committees of Duke University Research Ethics Committees of Peking University (IRB00001052-13074). The patients/participants provided their written informed consent to participate in this study.

## Author contributions

CC, YH, and TX conceived and designed the study. YH and TX participated in acquisition of the data and wrote the original draft. YH and CC contributed to data analysis. YH took charge of the submission. CC, XZ, YH, TX, QY, CP, LZ, and HC substantively revised the manuscript. All authors have read and approved the final manuscript.
